# Physical Activity Patterns among 9000 Pregnant Women in Poland: A Cross-Sectional Study

**DOI:** 10.3390/ijerph17051771

**Published:** 2020-03-09

**Authors:** Izabela Walasik, Katarzyna Kwiatkowska, Katarzyna Kosińska Kaczyńska, Iwona Szymusik

**Affiliations:** 1 Students Scientific Association at the 1st Department of Obstetrics and Gynecology, First Faculty of Medicine, Medical University of Warsaw, Plac Starynkiewicza 1/3, 02-015 Warsaw, Poland; drzewicaiza@wp.pl (I.W.); katarzyna.kwiatkowska@wp.pl (K.K.); 2 2nd Department of Obstetrics and Gynecology, The Center of Postgraduate Medical Education, Marymoncka st. 99/103, 01-813 Warsaw, Poland; 3 1st Department of Obstetrics and Gynecology, First Faculty of Medicine, Medical University of Warsaw, Plac Starynkiewicza 1/3, 02-015 Warsaw, Poland; iwona.szymusik@gmail.com

**Keywords:** physical activity, pregnancy, exercise, delivery

## Abstract

The aim was to analyze the knowledge and experience of women regarding physical activity during their latest pregnancy. An anonymous questionnaire was completed electronically, in 2018, by 9345 women who gave birth at least once, with 52% of the women having performed exercises during pregnancy. Physically non-active respondents suffered from gestational hypertension (9.2% vs. 6.7%; *p* < 0.01) and gave birth prematurely (9% vs. 7%; *p* < 0.01) to newborns with a low birth weight significantly more often (6% vs. 3.6%; *p* < 0.001). Physically active women delivered vaginally more often (61% vs. 55%; *p* < 0.001) and were more likely to have a spontaneous onset of the delivery as compared with non-active women (73.8% vs. 70.7% *p* = 0.001). The women who were informed by gynaecologist about the beneficial influence of physical activity during pregnancy exercised significantly more often (67% vs. 44% *p* < 0.001). In addition, 13% of the women felt discrimination due to their physical activity during a pregnancy, 22% of respondents’ physical activity was not accepted by their environment, and 39.1% of the women were told by others to stop physical exercise because it was bad for the baby’s health. Physical activity during pregnancy is associated with improved fitness, decreased pregnancy ailments occurrence, and therefore influences the course of pregnancy and delivery in a positive way.

## 1. Introduction

Physical activity during pregnancy has been widely discussed concerning the health and safety of the mother and infant. The American College of Obstetricians and Gynecologists (ACOG) created recommendations and encouraged all pregnant women to engage in moderate-intensity exercise for 150 min per week [1].

Physical activity maintains and improves cardiorespiratory fitness, reduces the risk of obesity and associated comorbidities, and results in greater longevity [1]. Performing exercises is an essential element of a healthy lifestyle, therefore, obstetricians and other obstetric care providers should encourage their patients to continue or to commence exercise as an important component of optimal health [1].

In the absence of contraindications, pregnant women should engage in a range of recreational activities, as exercise is not only safe but also associated with numerous maternal health benefits. Physical activity during pregnancy contributes to improved fitness and is associated with a decreased risk of preeclampsia [2] or gestational diabetes [3]. In addition, it helps to avoid excessive gestational weight gain [4] and improves the quality of life [5]. Recent studies have proven that exercising during pregnancy does not influence the risk of preterm labor or delivering a newborn small for gestational age [6,7,8,9,10,11]. However, the actual rate of women performing exercises during pregnancy in Poland is not known. 

The objective of this study was to assess the knowledge and experience of women regarding physical activity during their latest pregnancy.

## 2. Materials and Methods 

A cross-sectional survey was conducted. A self-composed questionnaire in Polish language was distributed via the internet between November and December 2018. The questionnaire was dedicated to pregnant Polish speaking women, regardless of inhabitancy. We distributed it by web pages and Facebook groups designed for pregnant women. No specific criteria, other than singleton delivery within the last year, were used for recruitment for the study. The survey was voluntary and anonymous; the survey did not contain any questions regarding personal data that would enable the identification of participants and only the authors of the study had access to the collected information. 

The questionnaire consisted of single or multiple-choice questions which evaluated the knowledge and experience related to physical activity during pregnancy. The survey was divided into four sections. The first part was comprised of sociodemographic data and knowledge regarding proper physical activity during pregnancy. In the second part, women were asked about their physical activity during pregnancy, including the type of exercise, frequency, and duration of training per week in each trimester. The third part referred to information about the delivery and the fourth part investigated the women’s opinion regarding family and social attitude to exercising during pregnancy.

Respondents were qualified as “physically active during pregnancy” if they performed exercises such as regular walks, marching, jogging, total body workout at a gym, swimming, yoga, pilates, fitness, exercise ball workouts, or home gymnastics. These kinds of activity are recommended by ACOG [1]. Exercises had to be done regularly (at least twice a week) and one training should last at least 15 min. Women who did not regularly perform any of the abovementioned activities or did not exercise at all were qualified as “physically inactive”. 

The inclusion criteria for the study were as follows: age > 18 years old, delivery ≥24 weeks of gestation maximum one year prior to completing the questionnaire, and a singleton live birth. Women in multiple pregnancies and those who miscarried or reported any major disabilities were excluded from the study. Only completely filled out questionnaires were taken into account. All the answers were checked for duplicates and no identical records were found. 

Body mass index (BMI) was defined as the body mass divided by the square of the body height. The following ranges for BMI were taken into account: underweight, <18.5 kg/m^2^; normal range, 18.5 to 24.99 kg/m^2^; overweight, 25 to 29.99 kg/m^2^; and obesity, >30 kg/m^2^. Gestational weight gain (GWG) was defined as the difference between the maternal weight at delivery and preconceptional weight [12]. The range of weight gain recommended by the Institute of Medicine for underweight (UW) was 12.5 to 18 kg, for normal weight (NW) women; 11.5 to 16 kg, for overweight women (OW); 7 to 11.5 kg and for obese women (OB); and 5 to 9 kg of weight throughout pregnancy [13]. Gestational hypertension (GH) and preeclampsia (PE) were defined according to the American College of Obstetricians and Gynecologists recommendations and gestational diabetes mellitus (GDM) according to the Polish Society of Obstetricians and Gynecologists recommendations [14,15]. Intrahepatic cholestasis of pregnancy (ICP) was diagnosed on the basis of symptoms of pruritus and elevated values of bile acids in the blood serum (>10 μmol/L). Women smoking cigarettes during 5 years before the pregnancy and during pregnancy, regardless of the smoking cessation, were considered as addicted to nicotine. A visual analogue scale [VAS] from 0 to 100 mm was used for labor pain assessment [16]. Preterm delivery was defined as one occurring at less than 37 completed weeks of gestation [17]. Low birth weight (LBW) was defined as neonatal birth weight less than 2500 g [18]. The study protocol obtained the approval of the Ethics Committee of the Medical University of Warsaw AKBE/293/2019. 

Data were expressed as absolute numbers and percentages or means and standard deviations. Statistical analyses were performed with the use of R software version 3.2.5 (R Foundation for Statistical Computing, Vienna, Austria). The χ2 or Fisher exact tests were used to compare categorical variables and Mann–Whitney U-test for continuous variables. All tests were two tailed and *p* < 0.05 was considered significant. 

## 3. Results

### 3.1. Study Population

A total of 9903 respondents filled out the questionnaire, among which 557 provided contradictory or mutually exclusive information or did not meet all the inclusion criteria. Eventually 9345 completed surveys were analyzed. Maternal characteristics of the study group are presented in Table 1. Most of physically active women were primiparous, had higher education, and lived in the cities with over 100,000 inhabitants.

### 3.2. Physical Activity during Pregnancy

The study group was further divided into respondent “physically active” and “non-active” during gestation. Among the 52% of women who performed exercises during pregnancy, the type of exercise included: 74.1% walking, 25% marching and brisk walking, 30.7% home gymnastics, 25% exercise ball workouts, 26% swimming, 5.2% running, and 1.75% total body workout at a gym. During the first and second trimester, 90% of the women exercised, while in the third trimester, almost 13% of respondents resigned from physical activity.

According to surveyed women, the most common reasons to be physically active during pregnancy included the following: improvement of general condition (67%), continuing pre-pregnancy physical activity (56.5%), the ability to recover faster after the forthcoming delivery (48.2%), and better preparation for the delivery (47.6%). Most women exercised regularly until the delivery date (47.5%) or until the last week before term (20.7%). However, they all decreased the intensity of exercising in the third trimester. In addition, 2.6% of the women ceased to exercise at the time of confirmation of pregnancy by a doctor. 

Most of physically active respondents exercised regularly for more than two years before pregnancy (54%) and 60% of non-active women did not exercise before pregnancy. The main reasons for the lack of physical activity during gestation were, the lack of interest in such activity (45%), feeling the lack of energy (40%), missing knowledge regarding proper exercises (34%), shortage of time (27%), and medical contraindications pointed by a physician (25%). There are a few contraindications to pregnancy physical activity defined by ACOG [1]. According to the ACOG list, 19% of women had existing contraindications to regular activity, among which the most common were cervical incompetence (6.9%), preeclampsia and pregnancy-induced hypertension (3.4%), and persistent second- and third-trimester bleeding (2.4%). However, as many as 38% of them ignored it and exercised without the doctor’s permission. 

### 3.3. Pregnancy Ailment

The occurrence of the most common discomforts of pregnancy, such as constipation, leg swelling, backache, mood swings, weakness, sleeping problems, decreased libido, heartburn, or calf cramps was investigated. The results showed that all of the analyzed pregnancy related ailments were significantly less often reported in the group of exercising respondents. The detailed results are presented in Table 2. 

### 3.4. Pregnancy Complications and the Course of Delivery

Table 3 presents the associations between exercising, gestational age at delivery, and newborns’ birth weight. Non-active respondents significantly more often gave birth prematurely (9% vs. 7%; *p* < 0.01) to newborns with LBW (5.95% vs. 3.6% *p* < 0.01). However, after the exclusion of preterm deliveries, the mean newborns’ birthweight did not differ between the groups (physically active, 3456 ± 454 g versus non-active, 3453 ± 474 g; *p* = 1). There were more vaginal deliveries among the women who exercised during pregnancy (61% vs. 55% *p* < 0.001). Moreover, physically active women were more likely to have a spontaneous onset of the delivery as compared to non-active women (73.8% vs. 70.7% *p* = 0.001). Exercising respondents assessed the delivery pain level as lower as compared with the non-active women (mean 7.81 ± 2.24 points vs. 8.14 ± 2.17 points respectively; *p* < 0.001). 

### 3.5. Sources of Information about Physical Activity during Pregnancy

Women were asked about the sources of their knowledge regarding the proper physical activity during pregnancy. Only one third of them claimed to gain information from obstetricians during antenatal counselling (28.1%), 69.2% of women sought information on the internet, and 30.3% at childbirth school meetings. However, only 66% of those who were attending childbirth schools admitted to receiving satisfying information about physical activity adequate for gestation during the meetings. Women who were informed by gynaecologist about the beneficial influence of physical activity during pregnancy exercised significantly more often (67% vs. 44%; *p* < 0.001) and 22.2% of all respondents claimed to be unable to identify reliable sources of information regarding exercise during gestation. 

### 3.6. Social Attitude towards Physical Activity during Pregnancy

The family and social attitude towards physically active pregnant women was assessed basing on respondents’ subjective views. The results indicated that 12.6% of women admitted to having felt discriminated by social opinion on exercising during pregnancy, 124 respondents performed exercises away from their homes to avoid being recognized and discriminated, 60% of women admitted to having heard negative opinions about physical activity during pregnancy, and only 29% of women experienced positive reactions from the society. The lack of acceptance was noticeable even among family members (31%) and 39.1% of respondents were advised not to exercise as it might be harmful for the baby.

## 4. Discussion

In the above presented study, almost 52% of respondents performed some kind of exercises during pregnancy. This result is concordant with previously published research [6,18,19,20,21,22]. Evenson et al. examined leisure activities during pregnancy among the U.S. population. They collected data was acquired over telephone interviews from 1979 pregnant women and 44,657 non-pregnant women 18 to 44 years of age. The prevalence of any leisure activity in the past month was 65.6% in pregnant women and 73.1% in non-pregnant women. The authors found that any leisure activity was related to higher education, younger age, and excellent or very good health [18]. In the presented study, physically active women were more likely to live in cities above 100,000 citizens and have higher education. In addition, primiparous women exercised significantly more often during pregnancy than multiparas. Ribeiro and Milanez reported similar outcomes. The knowledge of physical activity in pregnancy was significantly better in women with higher education [22].

The most common types of activity performed by women in our study were walking, marching and brisk walking, and home gymnastics. Our findings are concordant with other published research [6,18,21,23,24,25]. Evenson et al. found that the most common leisure activity for pregnant women was walking, followed by activities such as swimming laps and aerobics [18]. According to the review of the literature, walking provides an array of maternal and fetal health benefits and is minimally affected by commonly experienced barriers [23]. Vargas Nunes Coll et al. analyzed the most popular types of activity among the population of pregnant women and their changes throughout an 11 years period (2004 to 2015). Walking was the most common physical activity, followed by cycling and weight training. In the analyzed time period, the authors observed a decreasing interest in walking (from 77.2% in 2004 to 47.4% in 2015) and cycling (from 8% in 2004 to 3.1% in 2015) and an increasing engagement in weight training (from 6.1% in 2004 to 21% in 2015), water gymnastics (from 3.9% in 2004 to 9.7% in 2015), aerobics (from 2.7% in 2004 to 5.7% in 2015) and dancing (from 2% in 2004 to 4.9% in 2015) [24]. 

Half of respondents in our study did not perform any exercises during pregnancy, although only 25% of them had any contraindications to aerobic activity. Many studies indicate the most common barriers to exercise are at an intrapersonal level, i.e.; physical discomfort from nausea, fatigue, shortness of breath, heart burn, leg cramps, and body soreness [26,27,28,29]. Additional perceived barriers included the lack of time, lack of knowledge of adequate exercise during pregnancy [30], concerns about the possible injury [31], and lack of or incorrect information from healthcare providers [32,33,34,35]. Our results regarding the reasons not to exercise were similar to those presented by Haakstad et al. The researchers analyzed the barriers to start physical activity at 16 and 35 weeks of gestation. In the second trimester of pregnancy the intrapersonal barriers such as “... insufficient time”, “... I do not have the energy”, and “... the lack of interest (I would rather spend my time on other things)” were the most common. However, in the third trimester the health-related factors were perceived as the most important. Pelvic girdle pain and movement problems were the most frequently mentioned ones [36].

According to the presented study, physically active respondents were less likely to suffer from pregnancy-related ailments. Similar outcomes have been presented in a few studies. According to Arizabaleta et al.; exercising during gestation can improve the general quality of life. A supervised three month program of primarily aerobic exercises during pregnancy improved the health-related quality of life [5]. Pennick et al. found that acupuncture or exercise, tailored to the stage of pregnancy, significantly reduced the evening pelvic pain or lumbopelvic pain more than usual care alone [37]. The intensity of back and low back pain can increase with advancing pregnancy. Kihlstrand et al. showed that water gymnastics during the second half of pregnancy significantly reduced the intensity of these ailments [38]. Several studies confirmed that yoga could be the solution for low back pain treatment [39,40,41]. 

Previously published studies have found that moderate intensity exercise does not increase the risk of preterm delivery [6,7,8]. According to our study, non-active respondents gave birth prematurely significantly more often than regular exercisers. Owe et al. analyzed 61,098 singleton pregnancies enrolled between 2000 and 2006 in the Norwegian Mother and Child Cohort Study, conducted by the Norwegian Institute of Public Health. Data on self-reported exercise were collected by means of two questionnaires conducted at 17 and 30 weeks of gestation. The authors found that exercise performed during pregnancy shifted the gestational age distribution slightly upward, resulting in a reduced rate of preterm births and a slightly increased rate of post-term deliveries [9]. Inversely, sedentary lifestyle during pregnancy was related to an increased risk of preterm birth [6,7]. According to Aune et al.; higher leisure-time physical activity during pregnancy was associated with a significant decrease in the relative risk of preterm birth by 14%; for each increase in leisure-time physical activity during pregnancy by three hours per week, a 10% reduction in the risk of preterm birth was observed [10]. 

The impact of physical activity on the probability of vaginal labor has been extensively discussed in the literature [6,42,43,44,45,46,47]. Barakat et al. created a physical conditioning program for pregnant women that included a total of three 40 to 45 min sessions of exercise per week from six to nine weeks, until the end of the third trimester (38 to 39 weeks of gestation). The observed percentages of cesarean sections (15.9%) and instrumental deliveries (11.6%) in the exercising group were lower than in the control group (23% and 19.1%, respectively, *Z* = 2.73, *p* = 0.03, 1−β = 0.65) [44]. Similar results were published by Di Mascio et al.; who found that active women (aerobic exercise lasting 35 to 90 min three to four times per week) delivered vaginally more often (relative risk 1.09, 95% confidence interval 1.04 to 1.15) as compared with non-active women [6]. In our survey, physically active women also delivered vaginally significantly more often.

Observational studies on the association between maternal physical activity during pregnancy and neonatal birth weight have reported conflicting results. Some researchers found physical activity to be related to the decreasing risk of fetal macrosomia [48,49], whereas others found no significant association between exercises and neonatal birth weight [50,51]. According to the presented study, non-active respondents gave birth to newborns with low birth weight significantly more often, however, after exclusion of preterm deliveries the mean newborns’ birthweight did not differ between the groups. Physical activity during pregnancy has also been explored as a potential intervention to lower the risk of large-for-gestational age and macrosomia without increasing the risk of small-for-gestational age babies [8,52,53]. Harrod et al. found the exercise in leisure time physical activity during late (but not early) pregnancy to be associated with decreased neonatal adiposity, without significantly reduced neonatal fat-free mass [8]. Fat-free mass is believed to be related to fetal genetic growth potential, while fat mass is related to placental supply. The hypothesis of reducing the risk of fetal macrosomia is that physical activity during pregnancy could reduce fetal fat mass by increasing insulin sensitivity and by modulating glucose regulation [54,55]. Pastorino et al.; in a large cross-sectional cohort analysis of 72,694 individuals, found small but consistent inverse associations between maternal leisure time physical activity during late pregnancy and offspring birth weight. Each additional hour per week of moderate-to-vigorous physical activity in late pregnancy was associated with 6.4 g lower birth weight and 4% and 3% relative reductions in the risk of macrosomia and large-for-gestational age babies, respectively, without increasing the risk of small-for-gestational age newborns [56]. 

In the presented study, 12.6% of women admitted to feeling discriminated by society because of exercising during pregnancy. Marquez et al. found social support to be a powerful facilitator to start physical activity during pregnancy [28]. Many researchers claim that interpersonal barriers, including the lack of social support, could force women not to exercise during pregnancy [28,33,36,57,58,59,60,61]. Thornton et al. found that social support was the primary source of emotional, instrumental, and informational support for weight, diet, and physical activity-related beliefs and behaviors among pregnant women from Latin America. Unfortunately, almost 60% of our respondents admitted to having heard negative opinions about their physical activity during pregnancy [57]. Van Mulken et al. conducted a survey on 30 pregnant respondents who were discouraged from physical activity by people at work, the gym, and family. These women expressed that they were advised to “slow down” and were told that their current activities could be putting their baby’s health at risk [60]. In our study almost 40% of respondents were advised to stop exercising because it could be harmful for the infant’s health.

The strength of our study is its unique large group of pregnant respondents. To the best of our knowledge, no survey conducted on such a large population of women in Poland has been published, to date. The sample of respondents was diverse regarding sociodemographic characteristics such as age, education, inhabitancy, and numbers of deliveries. The anonymity and distribution of the questionnaire via internet could have promoted honesty of answers. The high response rates are certainly strengths of our study, however, some limitations need to be taken into consideration while interpreting the findings. Physical activity in our study was self-reported, which could result in an overestimation of the actual physical activity levels. All data were reported by respondents, and therefore were not verifiable. 

## 5. Conclusions

Physical activity during pregnancy is associated with improved fitness, decreased pregnancy ailments occurrence, and therefore influences the course of pregnancy and delivery in a positive way. Therefore, women with uncomplicated pregnancies should be encouraged to engage in physical activities before and during pregnancy. Providing women with reliable sources of information on physical activity during pregnancy is crucial. Further prospective research is needed to establish the effects of specific kinds of exercises on pregnancy complications and outcomes to elaborate effective behavioral counseling methods and optimal type, frequency, and intensity of exercise for women with different body mass index and different conditions during pregnancy.

## Figures and Tables

**Table 1:** Basic characteristics of the study group.

	Study Group N = 9345 % (N)	Physically Active N = 4892 % (N)	Physically Non-Active N = 4453 % (N)	*p*
Age				
<20	2.1 (199)	1.7 (83)	2.6 (116)	0.01
21–30	55.8 (5214)	53.2 (2603)	58.6 (2611)	<0.001
31–40	37.7 (3523)	40.5 (1983)	34.6 (1540)	<0.001
>40	4.4 (409)	4.6 (223)	4.2 (186)	0.4
Education				
Basic or vocational	5 (484)	3 (151)	8 (333)	<0.001
Secondary	28 (2606)	21 (1037)	35 (1569)	<0.001
Higher	67 (6255)	76 (3704)	57 (2551)	<0.001
Inhabitancy				
Countryside	25 (2353)	21 (1021)	30 (1332)	<0.001
City < 50000	18 (1712)	17 (826)	20 (886)	<0.001
City 50000–100000	13 (1167)	11 (558)	14 (609)	<0.001
City 100000–500000	18 (1661)	19 (926)	16 (735)	0.002
City > 500000	26 (2452)	32 (1561)	20 (891)	<0.001
Number of deliveries				
1	62 (5788)	65 (3193)	58 (2595)	<0.001
2	30 (2839)	28 (1365)	33 (1474)	<0.001
3 and more	8 (718)	7 (334)	9 (384)	0.001
Physical activity level before pregnancy				
I didn’t exercise	37 (3413)	15 (753)	60 (2660)	<0.001
Less than a year	15 (1423)	13 (641)	18 (782)	<0.001
1 or 2 years	14 (1312)	18 (857)	10 (455)	<0.001
More than 2 years	34 (3197)	54 (2641)	12 (556)	<0.001
Pre-pregnancy BMI				
Underweight	8 (762)	8 (405)	8 (357)	0.7
Normal	67 (6246)	70 (3435)	63 (2811)	<0.001
Overweight	18 (1647)	16 (770)	20 (877)	<0.001
Obese	7 (690)	6 (282)	9 (408)	<0.001
Gestational weight gain				
		UNDERWEIGHT		
adequate		35 (140)	36 (130)	0.9
inadequate		48 (196)	41 (146)	0.07
excessive		17 (69)	23 (81)	0.1
		NORMAL		
adequate		33(1130)	29(814)	<0.001
inadequate		31(1049)	28(797)	<0.001
excessive		37(1256)	43(1200)	0.2
		OVERWEIGHT		
adequate		29 (227)	26 (224)	0.4
inadequate		12 (95)	11 (97)	0.4
excessive		58 (448)	63 (556)	<0.001
		OBESE		
adequate		26 (74)	25 (102)	0.006
inadequate		27 (76)	23 (92)	0.07
excessive		47 (132)	52 (214)	<0.001

**Table 2:** Pregnancy ailments in the study group.

Ailments	Study Group N = 9345 %/N	Physically Active N = 4892 %/N	Physically Non-Active N = 4453 %/N	*p*
Constipation	36 (3368)	48 (1614)	52 (1754)	<0.001
Legs swelling	38 (3520)	47 (1637)	53 (1883)	<0.001
Backache	53 (4995)	51 (2532)	49 (2463)	0.001
Mood swings	47 (4426)	49 (2166)	51 (2260)	<0.001
Weakness	69 (6480)	50 (3224)	50 (3256)	<0.001
Sleeping problems	43 (3994)	51 (2034)	49 (1960)	0.018
Decreased libido	31 (2940)	47 (1374)	53 (1566)	<0.001
Heartburn	55 (5186)	49 (2549)	51 (2637)	<0.001
Painful legs cramps	35 (3300)	49 (1609)	51 (1691)	<0.001

**Table 3:** Pregnancy complications and course of delivery in the study group.

	Study Group N = 9345 %/N	Physically Active N = 4892 %/N	Physically Non-Active N = 4453 %/N	*p*
Gestational age at delivery (weeks)				
<37	8 (716)	7 (324)	9 (392)	<0.001
37–41	84 (7896)	85 (4158)	84 (3738)	0.2
>41	8 (733)	8 (410)	7 (323)	0.05
Newborn’s weight				
<1000	0.2 (19)	0.08 (4)	0.3 (15)	0.009
1000–1500	0.7 (66)	0.49 (24)	0.9 (42)	0.009
1500–2500	3.8 (356)	3.02 (148)	4.7 (208)	<0.001
2500–4000	82.6 (7722)	84.03 (4111)	81.1 (3611)	<0.001
4000–5000	12.4 (1155)	12.08 (591)	12.7 (564)	0.4
5000–6000	0.3 (27)	0.3 (14)	0.3 (13)	1
Diseases				
GH/PE	8 (739)	7 (329)	9 (410)	<0.001
Cholestasis	2 (166)	1.5 (73)	2 (93)	0.03
GDM treated with diet	8 (752)	8 (393)	8 (359)	0.9
GDMs treated with diet and insulin	11 (1010)	11 (521)	11 (489)	0.6
Type of delivery				
Vaginal delivery	58 (5404)	61 (2966)	55 (2438)	<0.001
Cesarean section	42 (3941)	39 (1926)	45 (2015)	<0.001
Delivery onset	N = 5404 *	N = 2966 *	N = 2438 *	*
Spontaneous	72 (3913)	74 (2190)	71 (1723)	0.010
Induced	27 (1465)	26 (762)	29 (703)	0.010
I don’t know	0.5 (26)	0.5 (14)	0.5 (12)	1
Episiotomy	*	*	*	*
Yes	64 (3438)	62 (1823)	66 (1615)	<0.001
No	36 (1955)	38 (1137)	34 (818)	<0.001
Don’t know	0.2 (11)	0.2 (6)	0.2 (5)	1
Epidural analgesia	*	*	*	*
Yes	21 (1117)	23 (668)	18 (449)	<0.001
No	67 (3636)	66 (1961)	69 (1675)	0.04
No, but I wanted to use epidural	12 (651)	11 (337)	13 (314)	0.09

* Group consist of women who delivered vaginally only.
